# Postnatal Growth Patterns in a Chilean Cohort: The Role of SES and Family Environment

**DOI:** 10.1155/2012/354060

**Published:** 2012-05-14

**Authors:** D. E. Kang Sim, M. Cappiello, M. Castillo, B. Lozoff, S. Martinez, E. Blanco, S. Gahagan

**Affiliations:** ^1^Division of Child Development and Community Health, University of California, San Diego, 9500 Gilman Drive No. 0927, La Jolla, CA 92093-0927, USA; ^2^Institute of Nutrition and Food Technology (INTA), University of Chile, El Líbano 5524, Santiago, Chile; ^3^Center for Human Growth and Development, University of Michigan, Ann Arbor 300 North Ingalls, 10th Floor, Ann Arbor, MI 48109-5406, USA

## Abstract

*Objective*. This study examined how family environmental characteristics served as mediators in the relationship between socioeconomic conditions and infant growth in a cohort of Chilean infants. *Methods*. We studied 999 infants, born between 1991 and 1996, from a longitudinal cohort which began as an iron deficiency anemia preventive trial. SES (Graffar Index), the Life Experiences Survey, and the Home Observation for Measurement of the Environment (HOME) were assessed in infancy. Using path analysis, we assessed the relationships between the social factors, home environment, and infant growth. *Results*. During the first year, weight and length gain averaged 540 grams/month and 6.5 cm/month, respectively. In the path analysis model for weight gain, higher SES and a better physical environment were positively related to higher maternal warmth, which in turn was associated with higher average weight gain. Higher SES was directly related to higher average length gain. *Conclusions*. In our cohort, a direct relationship between SES and length gain developed during infancy. Higher SES was indirectly related to infant weight gain through the home environment and maternal warmth. As the fastest growing infants are at risk for later obesity, new strategies are needed to encourage optimal rather than maximal growth.

## 1. Introduction

Infant growth can have important long-term health and developmental consequences. In the case of poor weight gain, cognitive development can be impaired [1, 2]. When infancy weight gain is rapid, risk for obesity and related conditions increases [3–8]. Whether or not infant weight gain relates to socioeconomic status depends on context. Infants gain less weight in resource poor settings in underdeveloped countries [9, 10]. In developed countries, lower socioeconomic status (SES) can relate to risk for poor infant growth through several possible mechanisms including higher rates of postpartum depression or family size [11–14]. On the other hand, a recent study in a developed country showed higher infant weight gain in lower SES individuals, largely explained by lower breast feeding rates [15]. Lastly, in some settings SES has not been found to influence infant ponderal growth [16]. Thus, it is important to understand the relationship of SES to infancy weight gain in a variety of contexts and identify factors that might mediate this relationship.

When the availability of breast milk or formula and weaning foods is adequate, the relationship between SES and infant weight gain might be explained by stressful circumstances and characteristics of the home environment and the parent-infant relationship. Low-SES families are more likely to experience uncontrollable life events and may have less healthy home environments for children [17], including lower quality stimulation available in the home for children [18]. Previous research has shown that negative circumstances associated with poverty and distress among parents can compromise parents' abilities to provide sensitive, involved, and consistent parenting [19–21]. Parenting style and infant growth have been most often explored in the context of failure to thrive, with harsh and neglectful parenting associated with poor infant growth [22].

Understanding linear growth as it relates to SES is also important, as height can be a marker of health risk. At the population level, adult height is socially determined [23, 24]. Height reflects prenatal and postnatal environments superimposed on genetic potential [25]. In addition, adult height shows an inverse association with cardiovascular [26, 27] and cardiorespiratory [27] disease, some cancers [28], and type 2 diabetes [29]. Socioeconomic gradients in height are also present in childhood and have been noted at the time of birth in representative samples [30]. A recent study in the UK that found differences in birth length based on SES did not show further differences in growth during infancy [30]. In contexts where infant nutrition is adequate, questions remain about whether or not social factors relate to linear growth during the first year.

In order to answer the question of whether or not postnatal growth was related to SES and how family environment might mediate these relationships, we used data on birth weight and length and 12-month weight, length, SES and variables from the Home Observation for Measurement of the Environment (HOME) [31] from a large, longitudinal cohort of Chilean infants. This allowed us to establish direct and indirect associations between SES, family factors and infant growth.

## 2. Methods

This study is a secondary data analysis to identify the relationship of socioeconomic status (SES) with ponderal (weight) and linear (length) growth and to identify family factors that mediate this relationship, in a cohort of Chilean infants during the first year of life. The infants were enrolled in a randomized controlled trial of iron to prevent iron deficiency anemia. The parent study is described first, followed by methods for the secondary data analysis.

## 3. Original Study

From 1991 through 1996, we recruited healthy low- to middle-income, urban Chilean infants with birth weights of ≥3 kg for a double-blind, randomized, controlled trial of iron supplementation between 6 and 12 months of age [32]. At the time in Chile, infant health was generally excellent. In fact, parasitic infections and generalized undernutrition were virtually absent. However, dietary iron deficiency was common, and iron supplementation during infancy was not routine. In the trial, infants were randomized to iron supplementation or usual nutrition at 6 months. All but 8 of the cohort were initially breast fed, but approximately one third were supplemented in the first 6 weeks with formula made from powdered full fat cow's milk. The cohort continues to be followed with waves of data collection at 5 y, 10 y, and 16 y. The protocols for the original infant study and follow-up studies have been approved annually by the Institutional Review Boards of the Universities of Michigan and Chile, and the University of California, San Diego.

## 4. Secondary Analysis of the Influence of SES on Growth and Mediating Factors

Of the 1657 infants who completed the preventive trial, 999 had complete data on all variables for this analysis. Those without complete data did not differ significantly from those included in this analysis by birth weight or length, sex, SES, or family factors.

### 4.1. Outcome Variable

Unclothed infant weight, using an electronic scale (to the nearest 0.01 kg), and length, on a recumbent length board (to the nearest 0.1 cm), were measured monthly in the first year by trained nurses [33]. Infant weight gain over the first year (grams per month) was calculated as: (weight (kg) at 1 year − birth weight (kg))/(age (days) ∗30.44 days/month). Infant length over the first year (grams per month) was calculated as (length (cm) at 1 year − birth length (cm))/(age (days) ∗30.44 days/month).

### 4.2. Variable of Interest

SES was measured using a modified Graffar index, which included 10 items concerning family size and structure, father presence, educational level of head of household, home ownership, and ownership of appliances [34]. For this analysis, the scale was dichotomized based on the median score of 27 (range of 16 to 47), with “0” referring to low SES and “1” referring to middle SES.

### 4.3. Social Factors

Mothers provided the following information at the 1-year assessment. Life stress was assessed using the Life Experiences Survey [35, 36] which included 22 items such as unstable employment of the head of household, serious family conflict, and serious illness of a relative, for a possible score of 0–22 (Cronbach alpha = 0.67). Maternal depression risk was assessed using the Center for Epidemiological Studies Depression Scale (CES-D) [37] scale; a score of 16 or greater indicates risk for depression. Adult-to-child ratio [14] served as a marker of family composition. This ratio was computed as the number of income-earning adults/number of children under age 15. This variable was trichotomized, with “0” referring to one adult to one child (ratio of 1), “1” referring to more adults than children (ratio greater than 1), and “−1” referring to more children than adults (ratio less than 1).

### 4.4. Other Mediating Factors

Mediating factors included variables representing the infant's family and home environments. The Home Observation for Measurement of the Environment scale (HOME), measured by direct observation, was used to evaluate the quality of the home environment for nurturing (Cronbach alpha = 0.80) [38]. In the US, 6 HOME subscales are used. In this Chilean sample, we identified 5 factors using exploratory factor analysis: “maternal warmth and emotional support,” “sibling participation,” “physical environment,” “father-infant interaction,” and “cognitive stimulation.” Table 1 displays Eigen values and items, with factor-item correlations of 0.35 or higher. “Father-infant interaction” and “cognitive stimulation” were not significantly associated with infant weight gain and were excluded from the final analysis in favor of a more parsimonious model. We tested additional covariates that might partially explain the relationship between SES and growth in weight and length. As the cohort was part of an IDA preventive trial, we tested whether IDA at one-year or random assignment to iron influenced the relationship between SES and growth in the first year. We tested the effect of gestational age and number of children on weight and length gain. Gestational age (in weeks) was assessed by the date of the last menstrual period; number of children was self-reported by the mother. Lastly, we tested whether 2 separate measures of breastfeeding were related to infancy weight and length gain: bottle supplementation at 6 weeks and still breastfeeding at 6 months. Both measures were self-reported by mothers during the infancy data collection period.

## 5. Statistical Analysis

Statistical analyses were conducted using SAS (9.2; Cary, NC) and SPSS (17; Chicago, IL). Descriptive statistics included means and frequencies (Table 2). We compared our sample (sample with complete data) to those excluded due to missing data, with *t-*test and chi-square analyses. We developed a path analysis model to assess the relationships between SES and correlated variables, adult-to-child ratio, and life stress, and infant weight gain and length gain, mediated by the family and home environment (sibling participation in child care, the physical home environment related to nurturing, and maternal warmth). Path analyses were performed using SAS PROC CALIS. Standardized regression coefficients and *t*-statistics were used to describe the final model; *t*-values >1.96 were statistically significant. Model fit was tested using the chi-square statistic (*χ* ^2^; *P* > 0.05). Goodness-of-fit indices included the root mean square error of approximation (RMSEA < 0.06), comparative fit index (CFI > 0.95), and standardized root mean square residual (SRMR < 0.03).

## 6. Results

Descriptive statistics are shown in Table 2. Infants were 53% male, averaged 3.5 kg and 50.6 cm at birth, gained on average 539 grams/month in the first year, and were at the 49th percentile for weight-for-age at 1 year. Infants' mean linear growth in the first year was 6.5 cm/month, and mean length-for-age percentile at 1 year was 48th percentile. Only 3 percent of the 1 year-olds had heights less than the 5th percentile. Most infants (59%) lived in households containing more children than adults. The life stress scores ranged from 0 to 14 and averaged 4.8 (2.7).  Table 3 shows additional descriptive information on the social variables, stratified by SES. Participants in the middle SES group, compared to those with low SES, had better optimal nurturing environment with higher maternal warmth and physical home environment, lower sibling participation in infant care, and fewer stressful events.

**Figure 1:**
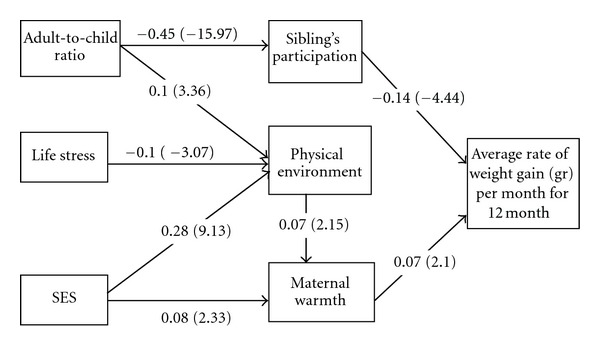
Standardized path coefficients (*t*-values) for a path model between SES and infant weight gain, mediated by home environment in 1-year-old Chilean infants (CFI = 0.97, SRMR = 0.02, RMSEA = 0.03). All paths are statistically significant (*P* < 0.05). SES significantly correlated with adult-to-child ratio and life stress. Model adjusted for direct relationship between number of children and average weight gain (*P* < 0.05).

Figure 1 shows the standardized path coefficients and respective *t*-values for the tested model. All paths shown are statistically significant associations. Fit indices indicated a good model fit (RMSEA = 0.03, CFI = 0.97, and SRMR = 0.02). SES was significantly correlated with adult-to-child ratio and life stress. Adult-to-child ratio was negatively associated with sibling participation (*B* = −0.45, *P* < 0.05), which negatively related to weight gain (*B* = −0.14, *P* < 0.05). SES (*B* = 0.28, *P* < 0.05) and adult-to-child ratio (*B* = 0.10, *P* < 0.05) were related to a more positive physical environment for nurturing, and higher life stress was related to a less positive physical environment (*B* = −0.10, *P* < 0.05). The physical environment indirectly related to weight gain through greater maternal warmth (*B* = 0.07, *P* < 0.05). SES was indirectly related to infant weight gain through a more positive physical environment (*B* = 0.28, *P* < 0.05) and greater maternal warmth (*B* = 0.08, *P* < 0.05).

We now examine the strength of the associations. Of the social factors, the strongest relationships were those between adult-to-child ratio and lower sibling participation in childcare and between SES and the physical environment for nurturing. The relationships between life stress and physical environment and adult-to-child ratio and physical environment were less robust, but significant. No direct associations between SES, adult-to-child ratio, and life stress and infant growth were found. We also tested potential covariates, including iron deficiency anemia, iron assignment, depression, gestational age, number of children, and bottle supplementation of breast feeding at 6 weeks and continued breast feeding at 6 months. With the exception of number of children, which had a direct inverse relationship with weight gain (*B* = −0.08, *P* < 0.05), these factors did not significantly contribute to the model and were omitted for parsimony. Sibling participation, maternal warmth, and number of children accounted for 3% of the variance in infant weight gain.

**Figure 2:**
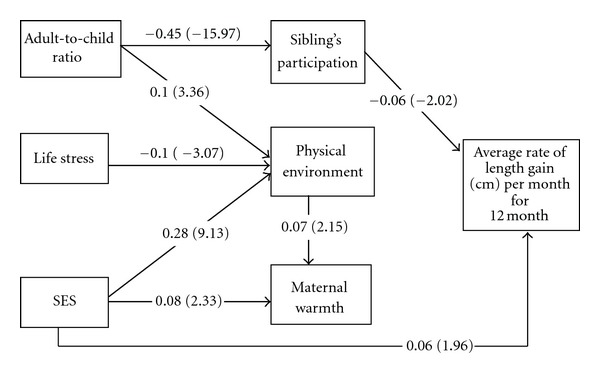
Standardized path coefficients (*t*-values) for a path model between SES and infant length gain, mediated by home environment in 1-year-old Chilean infants (CFI = 0.98, SRMR = 0.02, RMSEA = 0.03). All paths are statistically significant (*P* < 0.05). SES significantly correlated with adult-to-child ratio and life stress.

In examining infancy length gain (Figure 2), we found pathways identical to those associated with weight gain, with the exception that there was no relationship between maternal warmth and length gain. Additionally, SES was directly related to infancy length gain, rather than indirectly as was found for weight gain (*B* = 0.06, *P* = 0.05). This relationship was marginally significant, and the strength of the association was modest. As in the weight gain model, iron deficiency anemia, iron assignment, depression, gestational age, and bottle supplementation of breast feeding at 6 weeks and continued breast feeding at 6 month did not significantly contribute to the model and were omitted for parsimony. Number of children was directly and inversely related to infancy length gain (*B* = −0.07, *P* < 0.05). Sibling participation, number of children, and SES accounted for 1% of the variance in infant length gain.

## 7. Discussion

This study examined the relationship between SES and family factors and postnatal growth. Results showed that infant linear growth was directly related to SES, while ponderal growth was indirectly related to SES, mediated by the physical environment for nurturing and maternal warmth. In fact, the physical environment was a pivotal-mediating factor that linked the social variables to infant weight gain. This finding suggests that less strained financial circumstances provided a more supportive home environment. This, in turn, related to higher infant weight gain indirectly through maternal warmth.

Prior research has shown direct relationships between SES and birth length as well as SES and adult height [23, 24, 30]. However, in previous work, the infant SES-length relationship has been largely explained by the association already existing at birth [39]. This was not the case in our study, perhaps because infants weighing less than 3 kg at birth were excluded, resulting in less variation in birth length than would be found in a representative cohort. The relationship between SES and postnatal length gain was independent of birth length in our sample. This gradient was not mediated by the family or nutritional factors examined, suggesting that other unmeasured characteristics related to SES were responsible. It is highly unlikely that macronutrient factors were responsible for this finding, as none of the infants exhibited poor growth, almost all were initially breast fed, and supplemental milk was freely available from the Chilean National Health Service. Other potential explanations include micronutrient deficiencies or stressful circumstances affecting the hypothalamic-pituitary-adrenal axis.

In the context of this study of low- to middle-income, urban Chilean families, SES was related to infant weight gain. It is important to note that substantial public health programs in Chile led to a decline in infant mortality from 136.2/1000 live births in 1950 to 8.9/1000 live births in 2000. During this period, infant malnutrition was virtually eradicated through a supplemental milk program and a national breast feeding campaign that was highly successful [40]. In our study, the relationship between SES and infant growth was explained by family factors including family composition, the physical environment, and maternal warmth. All of the factors associated with more rapid weight gain are related to good nurturing. We also tested the role of breast feeding exclusivity and duration on these associations and found no effect. We suspect that the homogeneity of our sample in terms of high breast feeding rates minimized the effect of nutritional factors in our study. Another nutritional factor that could play a role is the timing of introduction of complementary foods, but we do not have data on this feeding practice. A recent study of infant growth in a multi-ethnic cohort in Amsterdam found higher growth rates in ethnic minority families that could not be entirely explained by different feeding practices [41]. Our study adds to the existing literature by supporting the role of psychosocial factors in infant growth. While there is a large literature-relating psychosocial factors to failure to thrive [22, 42], these characteristics are not always considered in infant growth research when failure to thrive is not the focus.

Our findings lead to a difficult question, how can optimal infancy growth be promoted in an era when failure to thrive is rare and risk for obesity is high. Historically, malnutrition presented serious risk for infants in Chile and had only recently been eradicated at the time our cohort participants were infants [40]. Currently in Chile, less than 2.9% of children under 6 years old are malnourished, and only 0.3% have moderate to severe malnutrition [40]. On the other hand, 16–20 years ago when our cohort participants were infants, the obesity epidemic was beginning in Chile but was not known or evident to families or even to health providers. In 2005, preschool age Chilean children had high obesity rates: 6% of 2-year olds, 11% of 3-year olds, and 14% of 4-year olds were obese. By young adulthood (17–24 years), the overweight and obesity prevalence was 24% in men and 28% in women [43]. In our study, the families with the most resources had infants with more rapid length and weight gain. We now know that rapid weight gain in infancy creates risk for later obesity [44, 45], which creates a conundrum for all involved. It appears that what would be considered optimal care is associated with increased risk for later obesity. Therefore, the fattest baby can no longer be viewed as the healthiest baby. While significant progress has been made in promoting breast feeding and encouraging families to delay supplemental bottles and complementary foods in Chile [40] and in many other settings [46, 47], it will be important to change perceptions of families about what a healthy baby looks like and what constitutes optimal growth. As these perceptions are highly linked with cultural norms, this will take a concerted effort on many fronts.

Some of this study's particular strengths are worth noting. This large cohort of infants was assessed at a university nutrition research center where the infants had detailed anthropometric measurement every month in the first year of life. In addition, data on SES, family factors and infant feeding were prospectively collected. Furthermore, most studies of SES and infant growth have been carried out in developed countries and may not relate to infants in other settings. Our study also has important limitations. The cross-sectional path analysis model cannot establish temporal precedence nor infer causality. It therefore must be considered to be hypothesis generating. Furthermore, findings about the SES infant-growth relationship from one context may not apply in other settings. Therefore, it may not be possible to generalize these findings to other cultures, rural settings, higher SES groups, or to other countries. Regarding generalizing to Chile in more recent years, however, it is important to note that infant growth rates in a more contemporary Chilean census, born between 2002 and 2004, are similar to those of our study for infants who weighted 3 kg or greater at birth [48].

## 8. Conclusion

In this developing country setting of rapid economic and nutritional transition, better circumstances related to higher infant growth. Prior to the onset of the global obesity epidemic, higher infant growth would have always been a sign of good parenting and prosperity and would have portended a survival advantage for the infant. In the current era, infants who are growing the fastest are at risk for developing obesity [45]. These findings emphasize the need for better understanding of explanatory factors related to infant growth. Future research should include longitudinal studies in a variety of settings that could identify possible causal elements including biological factors, such as genetics and the effects of the intrauterine environment, and environmental factors such as nutrition and the psychosocial environment.

## Figures and Tables

**Table 1:** Exploratory factor structures and item statistics: maternal warmth, sibling participation, and physical environment^a^.

Item content		Correlations	
	Maternal warmth	Sibling participation	Physical environment
Mother's voice conveys positive feelings toward child	45		
Mother caresses or kisses child at least once	45		
Mother spontaneously praises child at least twice	42		
Child's play environment is safe			74
When the child gets close to the mother, she welcomes, looks at, listens to, and is affectionate towards him/her	49		
The child is spontaneously taken up in the arms of his/her older siblings		76	
The child is spontaneously caressed, kissed, or tickled by older siblings at least 5 minutes of everyday		93	
The child is spontaneously conversed to in a directed and appropriate manner by older siblings		89	
The child is spontaneously incorporated into family activities by older siblings		84	
There are other people who are consistently important in the encouragement and care of the child		39	
The interior of the house has sufficient light and ventilation			46
The bedroom in the house is reasonably clean and orderly			55
With respect to the spare available space, there is sufficient room for the child to explore and crawl without danger			74

Eigen values	2.58	3.26	2.38

^a^Values are multiplied by 100 and rounded to the nearest integer. Only the factor items with correlations ≥ 35 are displayed.

**Table 2:** Descriptive statistics of Chilean infants (*n* = 999)^1^.

Birth weight (kg)^2^	3.5 (0.4)
Birth length (cm)^2^	50.6 (1.7)
Gestational age	39.4 (1.0)
Age at infancy evaluation	11.5 (0.4)
Weight-for-age percentile at 1 year^2^	48.7 (27.1)
Average weight gain (grams/month)	539.2 (84.2)
Length-for-age percentile at 1 year^2^	47.8 (25.8)
Average length gain (cm/month)	6.5 (0.3)
Bottle supplementation at 6 weeks	25.0
Still breastfeeding at 6 months	49.3
Number of siblings	2.1 (1.1)
Iron deficiency anemia in infancy	15.2
Iron supplemented	78.5
Maternal risk for depression	45.7
Life stress	4.8 (2.7)
Physical environment^3^	2.8 (1.3)
Maternal warmth^3^	3.4 (0.8)
Sibling participation^3^	3.9 (1.7)
Gender^2^	
Male	52.6
Female	47.4
SES	
Middle SES	52.7
Low SES	47.3
Adult-to-child ratio	
One-to-one ratio	26.4
More adults	15.1
More children	58.5

^1^Values are mean (SD) or %.

^2^Variable not included in the final model.

^3^Derived from HOME.

**Table 3:** Social factors by SES category.

	Middle SES	Low SES
Maternal warmth*	3.6 (0.8)	3.4 (0.8)
Sibling participation*	3.7 (1.7)	4.0 (1.6)
Physical environment*	3.2 (1.1)	2.4 (1.4)
More adults compared to children*	19.0%	10.8%
Stressful events*	4.5 (2.6)	5.0 (2.6)

*P < 0.05.
